# A Fully Automated Sequential-Injection Analyser for Dual Electrogenerated Chemiluminescence/Amperometric Detection

**DOI:** 10.1155/JAMMC/2006/67571

**Published:** 2006

**Authors:** Anastasios Economou, Maria Nika

**Affiliations:** 1 Laboratory of Analytical Chemistry Department of Chemistry Aristotle University of Thessaloniki Thessaloniki 54124 Greece

## Abstract

This work describes the development of a dedicated, fully automated sequential-injection analysis (SIA) apparatus suitable for simultaneous electrogenerated chemiluminescence (ECL) and amperometric detection. The instrument is composed of a peristaltic pump, a multiposition selection valve, a home-made potentiostat, a thin-layer electrochemical/optical flow-through cell, and a light detector. Control of the experimental sequence and simultaneous data acquisition of the light and the current intensities were performed in LabVIEW6.1. The CL reagents and the sample were first aspirated as distinct zones into the holding coil of the analyser and, then, delivered to the cell; during their travel, the individual zones mixed and the ECL reaction was initiated as soon as the mixed regents reached the cell. The utility of the analyser was demonstrated for the detection of oxalate and ${\text{H}}_{2}{\text{O}}_{2}$ based on their ECL reactions with $\text{Ru}{\left(\text{bpy}\right)}_{3}{}^{2}$ and luminol, respectively.


Hindawi Publishing Corporation
Journal of Automated Methods and Management in Chemistry
Volume 2006, Article ID 67571, Pages 1–9
DOI 10.1155/JAMMC/2006/67571

### A Fully Automated Sequential-Injection Analyser for Dual Electrogenerated Chemiluminescence/Amperometric Detection

**Anastasios Economou and Maria Nika**

*Laboratory of Analytical Chemistry, Department of Chemistry, Aristotle University of Thessaloniki, 54124 Thessaloniki, Greece*

Received 18 October 2005; Revised 29 December 2005; Accepted 16 January 2006

This work describes the development of a dedicated, fully automated sequential-injection analysis (SIA) apparatus suitable for simultaneous electrogenerated chemiluminescence (ECL) and amperometric detection. The instrument is composed of a peristaltic pump, a multiposition selection valve, a home-made potentiostat, a thin-layer electrochemical/optical flow-through cell, and a light detector. Control of the experimental sequence and simultaneous data acquisition of the light and the current intensities were performed in LabVIEW6.1. The CL reagents and the sample were first aspirated as distinct zones into the holding coil of the analyser and, then, delivered to the cell; during their travel, the individual zones mixed and the ECL reaction was initiated as soon as the mixed regents reached the cell. The utility of the analyser was demonstrated for the detection of oxalate and $H_2O_2$ based on their ECL reactions with $Ru(bpy)_3{}^2$ and luminol, respectively.

Copyright © 2006 A. Economou and M. Nika. This is an open access article distributed under the Creative Commons Attribution License, which permits unrestricted use, distribution, and reproduction in any medium, provided the original work is properly cited.

### 1. INTRODUCTION

Sequential-injection analysis (SIA) is the most recent version in the field of flow analysis techniques [1]. It is based on the sequential aspiration of the sample and reagent solutions into a holding coil as separate zones, followed by the delivery of the zones to the detector through a reaction coil. The advantages of SIA over conventional flow-injection analysis (FIA) are the reduced consumption of reagents, the convenience in varying the chemical and instrumental operating parameters, the ease of calibration, and the versatility and simplicity afforded by the single-channel manifold. The SIA principle is applicable to different direct detection schemes, such as UV-Vis spectrophotometry, IR spectrometry, atomic absorption spectroscopy, amperometry, and potentiometry [1]. In addition, it has been demonstrated that the SIA methodology is ideally suited to techniques that employ a preconditioning, pretreatment, or preconcentration stage of the sample before the actual measurement (such as enzymatic and immunological assays, solid phase extraction, and stripping voltammetry) [2].

Chemiluminescence (CL) is the phenomenon of production of light in the course of chemical reactions [3]. ECL is a special case of CL in which at least one of the species taking part (either directly or indirectly) in the light-emitting reaction is generated by a redox reaction on an electrode [4, 5]. Both CL and ECL detections are based on monitoring the intensity of light produced in the course of the light-emitting reaction and relating it to the analyte concentration in the sample. Although CL detection is ideally suited and widely applicable to FIA [6], it presents some problems when applied to SIA [7]. These problems are associated with the stopped-flow nature of SIA and the fact that CL reactions are fast; indeed, the CL reaction starts as soon as the sample zones are aspirated into the holding coil and, by the time the fully overlapped zones have reached the detector, much of the emitted light has been lost. This is the reason why CL detection is only occasionally used in SIA [1]. ECL is an elegant way to circumvent this difficulty since the actual CL reaction can be accurately controlled both temporarily and spatially: indeed, the required species that triggers the subsequent CL reaction can be generated *in situ* electrochemically within the detection cell so that the maximum of the emitted radiation is captured [4, 5]. Therefore, ECL, unlike conventional CL, is ideally suited as a detection technique in SIA. In addition, ECL presents some unique advantages compared to simple CL detection [4, 5]: (i) the rate and timing of the light-emitting reaction can be controlled by varying the potential of the generating electrode; (ii) *in situ* generation of reactants allows the use of otherwise unstable species; (iii) in many cases the cost of the analysis can be reduced by employing immobilised CL reagents that can be electrochemically regenerated; (iv) in some cases the manifold can be simplified by decreasing the number of reagents. The main drawbacks of ECL detection are the need for additional electrochemical instrumentation and the possibility that by-products of electrochemical reactions [4, 5] foul the electrode surface.

Although there is obviously great scope in applying ECL detection in SIA, surprisingly no applications of this combination in chemical analysis exist in the literature and this is partly explained by the lack of commercial instrumentation suitable for ECL detection in SIA. This article reports the construction of a fully automated SIA instrument for ECL detection in which the control and data acquisition are performed by the LabVIEW programming tool. The LabVIEW software package has been shown to be ideally suited to the task of automating analytical instrumentation [8–10]. The user-friendliness of the user interface (front panel of the programme) offers excellent flexibility and simplifies the selection of the operational parameters, the data acquisition, and the presentation and evaluation of the results. The construction of such dedicated instruments can circumvent the lack of commercial instrumentation for ECL studies and can extend the scope and applicability of SIA as an analytical technique. In addition, the apparatus is able to conduct spectroelectrochemical experiments by simultaneous recording of the current and light intensities. The utility of the instrumentation developed was demonstrated to detect oxalate and $H_2O_2$, through their ECL reactions with $Ru(bpy)_3^2$ [11] and luminol [5], respectively. In the present system, volumes of the sample solution (containing oxalate or $H_2O_2$) and the reagent solution (containing $Ru(bpy)_3^2$ or luminol, resp.) were sequentially aspirated as adjacent distinct zones in the holding coil of the analyser. Then, the flow was reversed and the zones were directed to the electrochemical/optical detector. During their travel, the zones dispersed, overlapped, and mixed. As soon as the mixed zones reached the detector, the ECL reaction was initiated and the light emitted was monitored.

### 2. EXPERIMENTAL

### 2.1. Reagents

The reagents were of analytical grade and were purchased from Merck (Darmstadt, Germany) unless stated otherwise. Deionised water was used throughout. A $10^{-3}$ mol L$^{-1}$ solution of $Ru(bpy)_3^2$ (from tris-(2,2'-bipiridyl)ruthenium(II) chloride hexahydrate, Aldrich) was prepared in 0.1 mol L$^{-1}$ phosphate buffer (pH 6) (Fluka). A $10^{-2}$ mol L$^{-1}$ solution of luminol was prepared in 0.1 mol L$^{-1}$ NaOH. Working solutions of $H_2O_2$ were prepared from 30% v/v $H_2O_2$ (Panreac) by appropriate dilution. Working solutions of oxalate were prepared from an aqueous $10^{-2}$ mol L$^{-1}$ solution of $Na_2C_2O_4$. The Nafion preparation was 5% w/v solution in a mixture of water and lower alcohols (Aldrich, St Louis, Missouri); the 2.5% working Nafion solution was prepared after dilution (1 : 1) of the commercial solution with absolute ethanol.

### 2.2. Immobilisation of $Ru(bpy)_3^{2+}$ in Nafion

The platinum working electrode was polished with 0.2 $\mu$m alumina and rinsed with ethanol and water. A 25 $\mu$L drop of the 2.5% Nafion solution was placed on the surface of the electrode and allowed to dry for 10 min in ambient temperature and further treated with hot air from a heat gun for another 2 min. The electrode was sequentially immersed for 1 hour in a 0.1 mol L$^{-1}$ $H_2SO_4$ solution, for 30 min in a $10^{-3}$ mol L$^{-1}$ $Ru(bpy)_3^{2+}$ solution prepared in 0.1 mol L$^{-1}$ $H_2SO_4$, and, finally, for 30 min in a 0.1 mol L$^{-1}$ $H_2SO_4$ solution.

### 2.3. Instrumentation

The SIA analyser was designed and constructed entirely in-house; a schematic diagram of the complete SIA instrument is illustrated in Figure 1(a). Solution aspiration and delivery were accomplished by means of a peristaltic pump (Gilson Minipuls 3, France). A 10-port valve (Vici-Valco, Switzerland) served as a selection valve. The CL detector was a miniature Hamamatsu H6780 photomultiplier tube (PMT) operating at 600 V (Hamamatsu Photonics, Japan). The combined electrochemical/optical thin-layer flow cell was designed and constructed in-house and was placed in front of the detector in a light-tight box (as shown in Figure 1(b)). The working electrode was a platinum disk (2 mm in diameter), the reference electrode was a home-made, gel-based Ag/AgCl, and the counter electrode was a stainless steel tube also serving as the solution outlet. The thin-layer flow channel was defined by a 0.5 mm Teflon spacer placed between the two parts forming the cell. The electrodes were connected to a home-made potentiostat equipped with external terminals for potential input and current output. All the connecting tubing was PTFE 0.75 mm i.d. (Jour Research, Sweden). The pump, valve, and potentiostat were interfaced to a Pentium PC through a multifunction interface card (6025 E PCI, National Instruments, Tex). The pump made use of two TTL signals (one for on/off and a second for the forward/reverse direction) and one DAC channel (for speed control). The speed of the pump was calibrated in mL min$^{-1}$. The valve was controlled by BCD code that allowed selection of the port and reading back of the port selected. This operation required 14 TTL lines. The control of the potential of the working electrode necessitated the use of one DAC channel, connected to the potential input terminal of the potentiostat. The current was recorded by means of an ADC line connected to the current output terminal of the potentiostat while the emitted light (i.e., the voltage output from the PMT) was sampled directly by a second ADC channel.

### 2.4. Software

Programming was accomplished in LabVIEW 6.1 (National Instruments). The computer screen displayed two so-called "front panels" as illustrated in Figure 2. The front panel in Figure 2(a), called "sia_ecl.vi," is the front panel of the main control programme of the analyser. It contains (i) a control in

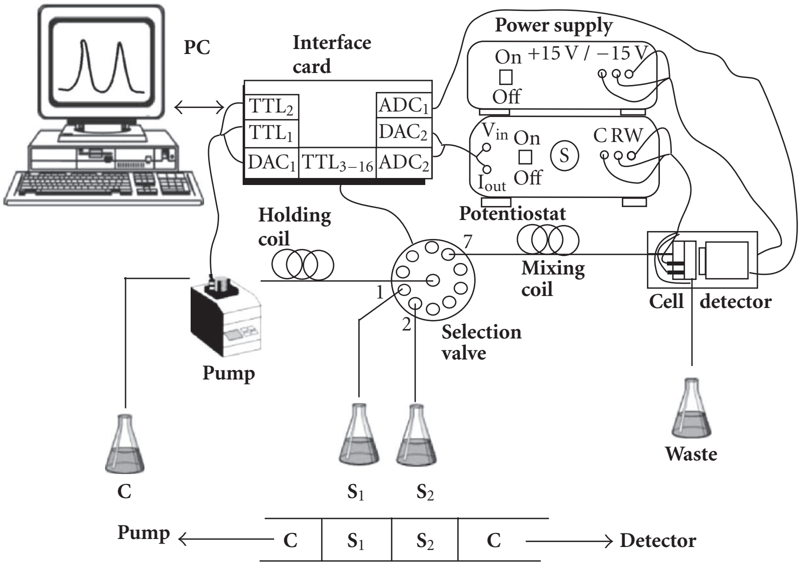

(a)

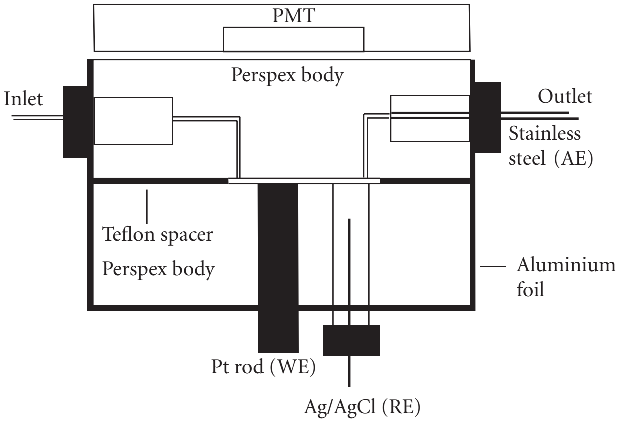

(b)

Figure 1: (a) The experimental configuration of the SIA apparatus for ECL detection $S_1$ = sample containing oxalate or $H_2O_2$, $S_2$ = 5 mmol $L^{-1}$ $Ru(bpy)_3^{2+}$ in phosphate buffer (pH 6) or 0.1 mmol $L^{-1}$ luminol in 0.01 mol $L^{-1}$ NaOH, C = carrier (KCl 0.01 mol $L^{-1}$); (b) schematic diagram of the electrochemical/optical flow-through cell.

the form of table (indeed a multidimensional array) where all the parameters of every experimental step (pump speed, flow direction, time of operation, valve position, and potential applied) are typed; (ii) a control in the form a single-column table (a single-dimensional array) where the proper sequence of steps to be used in the actual analysis is typed; (iii) a numerical control where the number of repeated injections is typed; and (iv) an indicator in the form of a column of LEDs (a one-dimensional array), one of which is flushing to indicate the step in the sequence that is executed at any one time. Part of the block diagram of this application is illustrated in Figure 3(a).

The front panel in Figure 2(b), called “ecl.vi,” is the front panel of the programme that performs the data acquisition from the PMT (light intensity) and from the potentiostat (current intensity). This programme is composed of (i) an indicator in the form of a strip chart that records the CL intensity with respect to time; (ii) another indicator in the form of a strip chart that records the current intensity with respect to time; (iii) a STOP control that, when pressed, stops the data acquisition and opens a file dialogue prompting to save the data in ASCII format (as a multidimensional array of points-time, current, CL intensity); and (iv) a control of the current sensitivity of the potentiostat which is used to calibrate the current output from the potentiostat. The block diagram of this application is illustrated in Figure 3(b).

Cyclic voltammetry experiments were conducted with a programme developed in LabVIEW, as reported elsewhere [8].

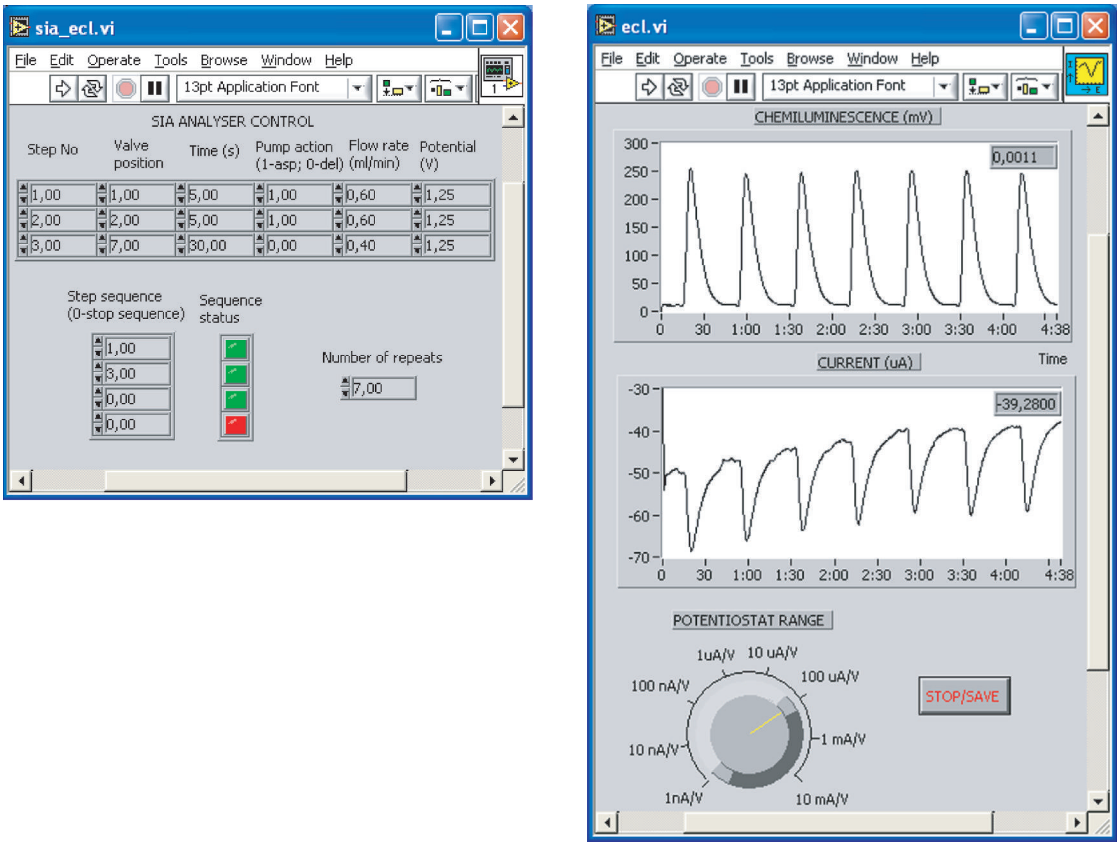

(a) (b)

Figure 2: (a) The front panel of the control programme developed for SIA; (b) the front panel of the data acquisition programme developed for ECL/amperometric detection.

### 2.5. Results and discussion

The ECL reaction between oxalate and $Ru(bpy)_3^{2+}$ is one of the most widely studied ECL reactions and occurs according to the following sequence of reactions [11]. It is initiated by the oxidation of $Ru(bpy)_3^{2+}$ to $Ru(bpy)_3^{3+}$ on the working electrode:

$$Ru(bpy)_3^{2+} \longrightarrow Ru(bpy)_3^{3+} + e^-. \quad (1)$$

$Ru(bpy)_3^{3+}$ reacts with oxalate to form the excited species $Ru(bpy)_3^{2+*}$:

$$\begin{gathered} Ru(bpy)_3^{3+} + C_2O_4^{2-} \longrightarrow Ru(bpy)_3^{2+} + C_2O_4^{-\bullet}, \\ C_2O_4^{-\bullet} \longrightarrow CO_2^{-\bullet} + CO_2, \\ Ru(bpy)_3^{3+} + CO_2^{-\bullet} \longrightarrow Ru(bpy)_3^{2+*} + CO_2, \\ Ru(bpy)_3^{2+} + CO_2^{-\bullet} \longrightarrow Ru(bpy)_3^{+} + CO_2, \\ Ru(bpy)_3^{3+} + Ru(bpy)_3^{+} \longrightarrow Ru(bpy)_3^{2+*} + Ru(bpy)_3^{2+}. \end{gathered} \quad (2)$$

Finally, the excited $Ru(bpy)_3^{2+*}$ species returns to its ground state $Ru(bpy)_3^{2+}$ by emitting light while the CL intensity is monitored and related to the oxalate concentration in the sample:

$$Ru(bpy)_3^{2+*} \longrightarrow Ru(bpy)_3^{2+} + h\nu \, (620\,\text{nm}). \quad (3)$$

Therefore, the whole sequence of reactions is triggered by the oxidation of $Ru(bpy)_3^{2+}$ to $Ru(bpy)_3^{3+}$ (reaction (1)) and finishes with the regeneration of $Ru(bpy)_3^{2+}$ (reaction (3)) which, thus, serves as a quasi-catalyst. There are three ways to electrochemically produce $Ru(bpy)_3^{3+}$ from $Ru(bpy)_3^{2+}$ [11]. The first method is the *ex situ* (external) preparation of the $Ru(bpy)_3^{3+}$ reagent by bulk oxidation of $Ru(bpy)_3^{2+}$ under coulometric conditions in a separate electrochemical cell; the resulting $Ru(bpy)_3^{3+}$ can be subsequently used to trigger the CL reaction. The second strategy is the *in situ* solution generation scheme in which $Ru(bpy)_3^{3+}$ is formed within the electrochemical/optical detection cell by oxidation of a $Ru(bpy)_3^{2+}$ solution. The *in situ* generation mode is advantageous, especially when combined with flow systems and it has been extensively used for the determination of reducing species in flow-injection analysis (FIA) [11]. The last scheme for generating $Ru(bpy)_3^{3+}$ is the *in situ* immobilisation generation of $Ru(bpy)_3^{3+}$. This mode involves immobilisation of $Ru(bpy)_3^{2+}$ on the working electrode which, upon application of an oxidising potential, is oxidised to

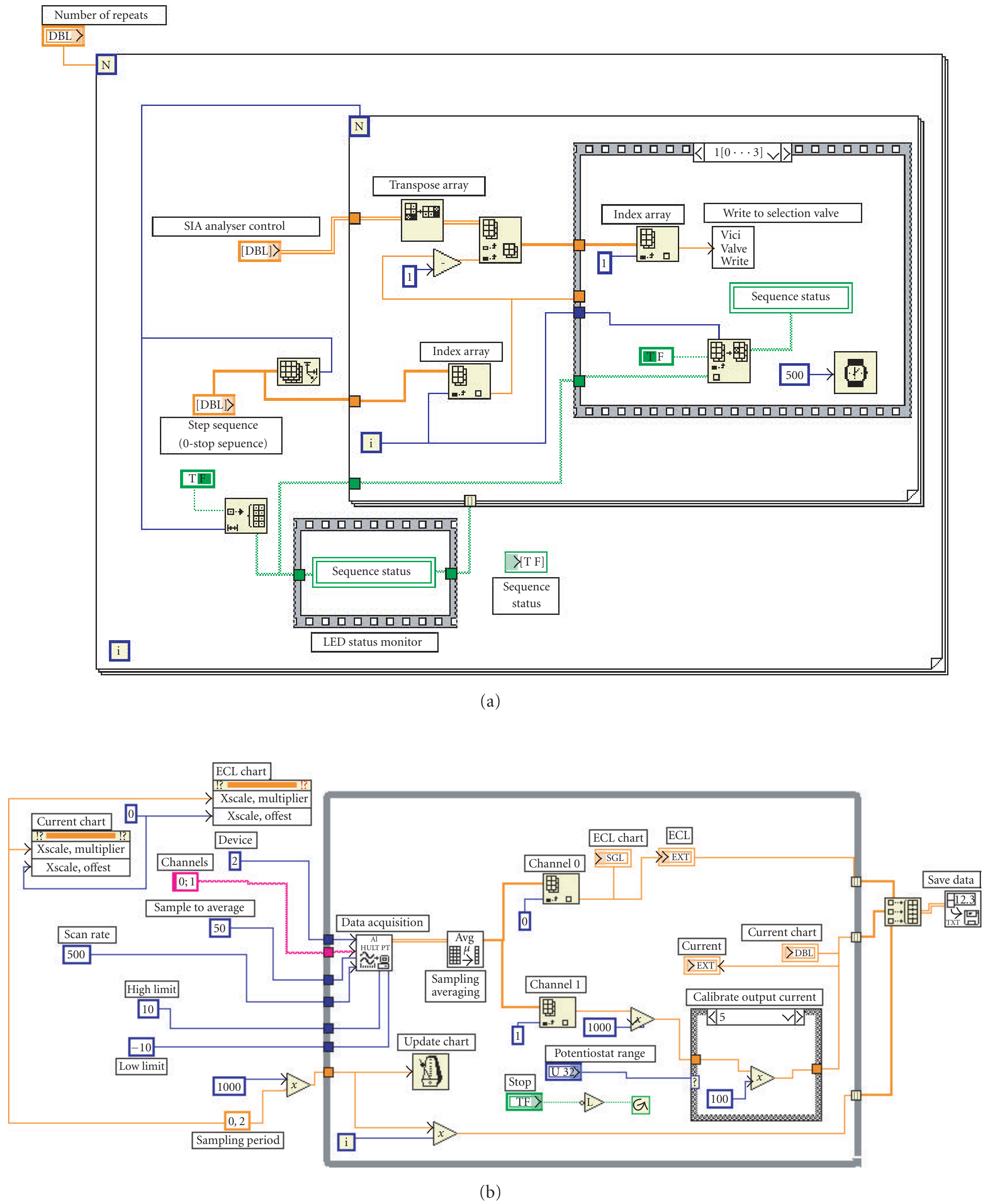

Figure 3: (a) Part of the block diagram of the control programme developed for SIA whose front panel is illustrated in Figure 2(a); (b) the block diagram of the data acquisition programme developed for ECL/amperometric detection whose front panel is illustrated in Figure 2(b).

$Ru(bpy)_3^{2+}$ and converted back to $Ru(bpy)_3^{2+}$ by the analyte. Thus the ruthenium complex is continuously cycled between its oxidised and reduced forms (reactions (1) and (3)) within the immobilising media without need to replenish it; this way of producing $Ru(bpy)_3^{3+}$ is especially suitable for the fabrication of ECL sensors [4, 5, 11].

The system was first applied to study the electrochemical behaviour of $Ru(bpy)_3^{2+}$ by performing cyclic voltmammetry from 0 to +1 V in order to access the feasibility of the *in situ* solution electrochemical generation of $Ru(bpy)_3^{3+}$. For that purpose, a volume of the 1 mmol $L^{-1}$ of $Ru(bpy)_3^{2+}$ solution in phosphate buffer (pH 6) was aspirated into the

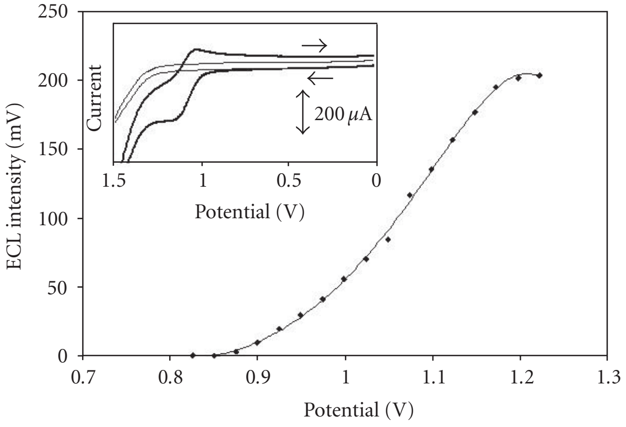

Figure 4: (inset) Cyclic voltammogram of a solution containing 1 mmol $L^{-1}$ $Ru(bpy)_3^{2+}$ in phosphate buffer (pH 6); ECL intensity (expressed as the SIA-ECL peak height) for the analysis of a sample containing 0.5 mmol $L^{-1}$ oxalate as a function of the potential applied to the working electrode.

holding coil and delivered to the flow cell. As soon as the sample plug reached the flow cell, the flow was stopped and the respective cyclic voltammogram was recorded in quiescent solution (inset in Figure 4, thick trace). Also, a blank cyclic voltammogram was recorded in the same way by aspirating pure buffer instead of the $Ru(bpy)_3^{2+}$ solution (inset in Figure 3, thin trace). The forward scan of the cyclic voltammogram indicated that the oxidation current increased sharply at potentials more positive than +1 V and exhibited an anodic peak at +1.17 V which was due to the electrochemical oxidation of $Ru(bpy)_3^{2+}$ to $Ru(bpy)_3^{3+}$ according to reaction (1). The reverse cathodic scan revealed a cathodic peak which was due to the reduction of the $Ru(bpy)_3^{3+}$ generated in the first scan back into $Ru(bpy)_3^{2+}$. This behaviour was consistent with previous studies of the $Ru(bpy)_3^{2+}$ species on platinum electrodes in the batch mode [12] and demonstrates that it is possible to achieve *in situ* generation of $Ru(bpy)_3^{3+}$ from a solution of $Ru(bpy)_3^{2+}$ in the combined electrochemical/optical cell.

Then, the possibility was investigated of detecting and determining oxalate by means of *in situ* electrochemically generated of $Ru(bpy)_3^{3+}$ from the solution phase under the typical conditions prevailing in SIA. For this purpose, the experimental sequence shown in Table 1 was programmed in the analyser.

According to this scheme, a zone of the oxalate solution was aspirated into the holding coil followed by a zone of the $Ru(bpy)_3^{2+}$ solution. Then, the two adjacent zones were directed to the detector through the mixing coil. During their travel, the two zones merged so that a solution containing both oxalate and $Ru(bpy)_3^{2+}$ solution reached the cell whose potential was maintained at +1.25 V and the ECL reaction took place. The ECL was recorded at different potentials applied to the working electrode in the range 0.825–1.225 V (Figure 4, main curve). This study indicated that no light was generated at potentials less than +0.85 V while the ECL gradually started to increase at potentials more positive than +0.9 V and eventually reached a plateau at +1.175 V. This behaviour was consistent with the results of the cyclic voltammetry experiments discussed in the previous paragraph and analogous experiments in the FIA mode [13]. No $Ru(bpy)_3^{3+}$ was formed at potentials lower than +0.85 V while more $Ru(bpy)_3^{3+}$ was generated as the potential of the electrode was gradually shifted to more positive values until the diffusion-limited potential region was reached. These results suggest that it is possible to detect oxalate by means of its ECL reaction with $Ru(bpy)_3^{2+}$ with in *situ* electrochemical solution generation of $Ru(bpy)_3^{3+}$ in the SIA mode provided that a sufficiently oxidizing potential (more positive than +1.2 V) is applied.

Then, the possibility for quantitative determination of oxalate was investigated by carrying out a calibration procedure in the range 5 $\mu$mol $L^{-1}$–5 mmol $L^{-1}$ of oxalate. Typical ECL peaks are illustrated in Figure 5 where an ECL signal can be noticed for the blank solution as reported earlier [11]. The corresponding calibration curve (Figure 5, inset) exhibits a wide linear range in the interval 10 $\mu$mol $L^{-1}$–2 mmol $L^{-1}$, a feature inherent in many CL and ECL detection schemes. The calibration can be expressed by the equation

$$\mathrm{ECL} = (366679 \pm 2645)c(\mathrm{oxalate}) + (4 \pm 2), \qquad R^2 = 0.99, \tag{4}$$

where ECL is the ECL intensity (mV); $c$(oxalate) is the oxalate concentration (mol $L^{-1}$).

An example of the *in situ* immobilisation generation scheme of $Ru(bpy)_3^{3+}$ from $Ru(bpy)_3^{2+}$ immobilised on a Nafion film is illustrated in Figure 6 where the ECL and amperometric signals of oxalate are compared. The apparatus allows the simultaneous recording of both current and light intensities, thus enabling spectroelectrochemical investigation *in situ* [14]. In this example, it is clear that the ECL response exhibits a more stable baseline as well as a better signal-to-noise ratio than the amperometric response.

The scope of the present system can be extended to encompass alternative widely used ECL reactions. As an example, the determination of $H_2O_2$ by means of its ECL reaction with luminol was considered. A simplified mechanism of this complex reaction is illustrated in Scheme 1 [15]. It is generally acknowledged that the sequence starts with the electrochemical oxidation of luminol to a diazo compound in alkaline media. Further chemical oxidation of the diazo compound produces 3-aminophthalate in its excited state which relaxes to its ground state with the simultaneous emission of light. The chemical oxidation step can be promoted in the presence of $H_2O_2$, hence the potential for analytical applications of this reaction.

In Figure 7, SIA-ECL peaks are recorded in the presence and absence of $H_2O_2$ with the electrode potential maintained at +0.65 V; this potential was selected as the optimum in an experimentally derived curve of ECL *versus* electrode potential similar to that of Figure 4. A small ECL signal was observed in the absence of $H_2O_2$ probably due to the weak oxidation of the diazo compound by dissolved oxygen [15]. However, in the presence of $H_2O_2$, the ECL signal was greatly

Table 1: Step sequence for the determination of oxalate and $H_2O_2$ by SIA-ECL ($S_1$ = oxalate or $H_2O_2$, $S_2$ = 5 mmol $L^{-1}$ $Ru(bpy)_3^{2+}$ in phosphate buffer (pH 6) or 0.1 mmol $L^{-1}$ luminol in 0.01 mol $L^{-1}$ NaOH).

| Step | Duration (s) | Pump speed (mL $min^{-1}$) | Pump status | Valve position | Electrode potential (V) | Step description |
|---|---|---|---|---|---|---|
| 1 | 5 | 0.6 | Aspirate | 1 | * | Aspirate zone of $S_1$ into holding coil |
| 2** | 5 | 0.6 | Aspirate | 2 | * | Aspirate zone of $S_2$ into holding coil |
| 3 | 40 | 1.2*** | Deliver | 7 | * | Deliver zones to detector through mixing coil |

* +1.25 and +0.65 V for oxalate and $H_2O_2$ detection, respectively.
** This step is omitted in the case of the Nafion electrode modified with $Ru(bpy)_3^{2+}$.
*** The flow rate in the case of the Nafion electrode modified with ($Ru(bpy)_3^{2+}$) was 0.4 mL $min^{-1}$.

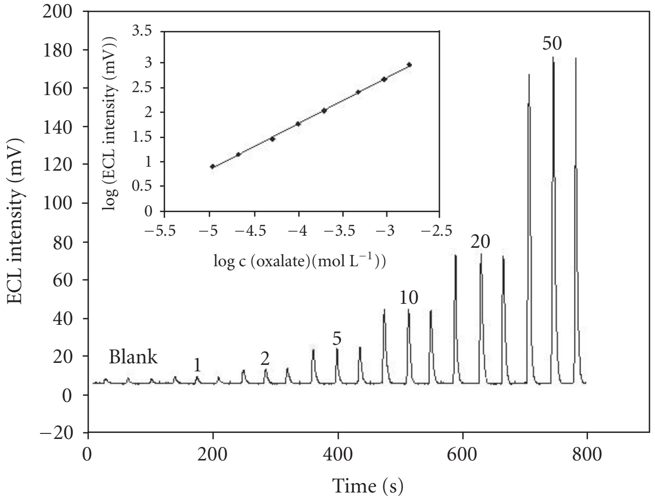

Figure 5: (a) SIA-ECL peaks for increasing concentrations of oxalate (concentrations above the peaks expressed in $10^{-5}$ mol $L^{-1}$); (b) log-log calibration curve in the range 10 $\mu$mol $L^{-1}$ to 2 mmol $L^{-1}$ (conditions as in Table 1).

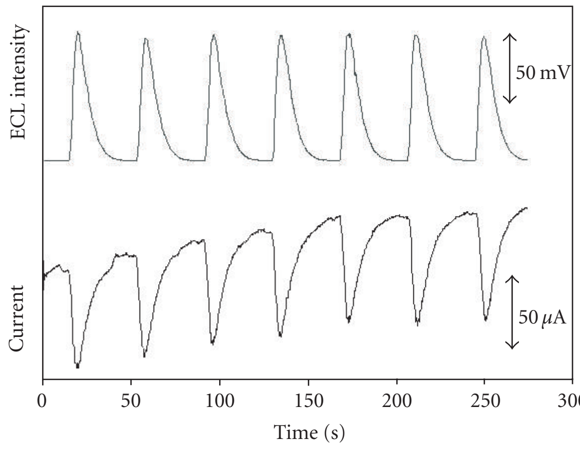

Figure 6: SIA-ECL and SIA-amperometric peaks of a solution containing 1 mmol $L^{-1}$ oxalate on the $Ru(bpy)_3^{2+}$-modified Nafion electrode (in this case only steps 1 and 3 in the sequence in Table 1 were used).

enhanced, suggesting that it is possible to use this reaction for the determination of either $H_2O_2$ itself or of species that take part in a reaction in which $H_2O_2$ is formed (i.e., substrates of enzymatic reactions catalysed by oxidases or dehydrogenases).

### 3. CONCLUSIONS

This work has, for the first time, demonstrated that it is possible to utilise ECL detection in SIA. For this purpose, a dedicated FIA-ECL analyser with provision for dual CL/amperometric detection has been designed and constructed from commercial components and a suitable software programme was developed for control and data acquisition. The main advantage of SIA-ECL is the possibility to conduct the analysis even for fast light-emitting reactions as a result of the *in situ* triggering of the reaction in the detection cell, unlike conventional SIA-CL. This technique allows a considerable reduction in the consumption of sample and expensive CL reagents (such as $Ru(bpy)_3^{2+}$ salts). It provides a high level of automation and versatility. It minimises electrode fouling because only minute volumes of reagents are injected unlike conventional FIA where reagents are continuously pumped through the cell. The apparatus developed in this work can also provide simultaneous CL and amperometric measurements. This provides a useful tool for

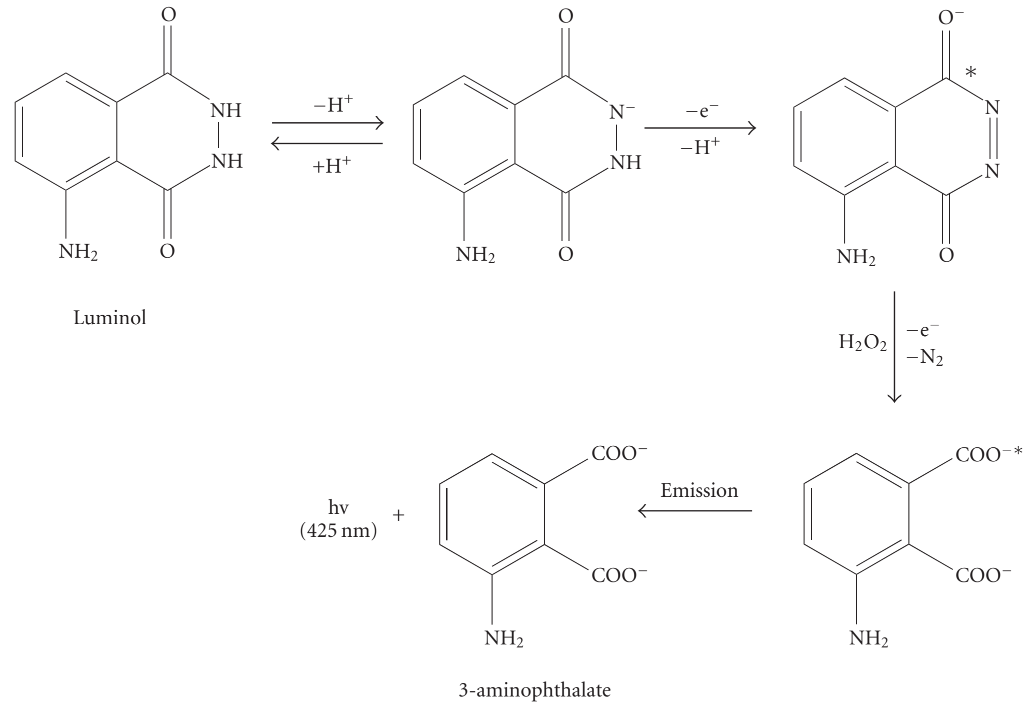

Scheme 1: Simplified mechanism of the ECL oxidation of luminol.

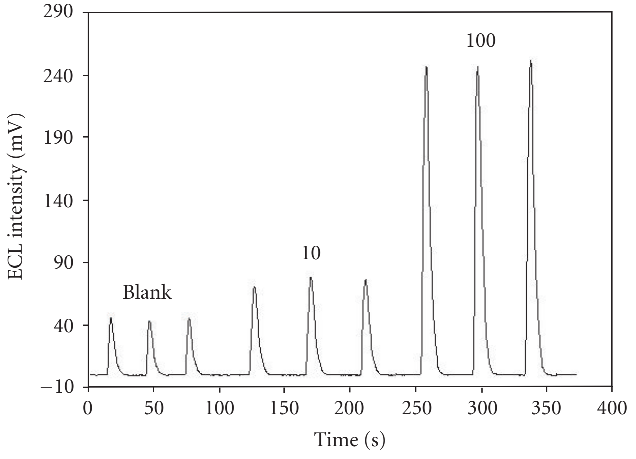

Figure 7: (a) SIA-ECL peaks for increasing concentrations of $H_2O_2$ using the ECL reaction of luminol oxidation ($H_2O_2$ concentrations above the peaks expressed in $\mu$mol $L^{-1}$) (conditions as in Table 1).

spectroelectrochemical studies. Finally, an important property of SIA, namely, its ability for complex sample handling prior to the actual detection, can be further explored.

### REFERENCES

[1] C. E. Lenehan, N. W. Barnett, and S. W. Lewis, "Sequential injection analysis," *The Analyst*, vol. 127, no. 8, pp. 997–1020, 2002.

[2] A. Economou, Sequential-injection analysis (SIA): a useful tool for on-line sample handling and per-treatment. *Trends in Analytical Chemistry*, vol. 24, pp. 416–425, 2005.

[3] A. M. García-Campaña and W. R. G. Baeyens, Eds., *Chemiluminescence in Analytical Chemistry*, Marcel Dekker, New York, NY, USA, 2001.

[4] A. W. Knight, Electrogenerated Chemiluminescence. *Chemiluminescence in Analytical Chemistry*, A. M. García-Campaña and W. R. G. Baeyens, Eds., pp. 212–246, Marcel Dekker, New York, NY, USA, 2001.

[5] K. A. Fähnrich, M. Pravda, and G. G. Guilbault, "Recent applications of electrogenerated chemiluminescence in chemical analysis," *Talanta*, vol. 54, no. 4, pp. 531–559, 2001.

[6] A. C. Calokerinos and L. P. Palilis, "Chemiluminescence in flow injection analysis," in *Chemiluminescence in Analytical Chemistry*, A. M. García-Campaña and W. R. G. Baeyens, Eds., pp. 321–348, Marcel Dekker, New York, NY, USA, 2001.

[7] N. Pizà, M. Miró, J. M. Estela, and V. Cerdà, "Automated enzymatic assays in a renewable fashion using the multisyringe flow injection scheme with soluble enzymes," *Analytical Chemistry*, vol. 76, no. 3, pp. 773–780, 2004.

[8] A. Economou, G. J. Volikakis, and C. E. Efstathiou, "Virtual instrumentation for electro-analytical measurements," *Journal of Automated Methods & Management in Chemistry*, vol. 21, no. 2, pp. 33–38, 1999.

[9] A. Economou and A. Voulgaropoulos, "LabVIEW-based sequential-injection analysis system for the determination of trace metals by square-wave anodic and adsorptive stripping voltammetry on mercury-film electrodes," *Journal of Automated Methods & Management in Chemistry*, vol. 25, no. 6, pp. 133–140, 2003.

[10] A. Economou, S. D. Bolis, C. E. Efstathiou, and G. J. Volikakis, "A "virtual" electroanalytical instrument for square wave voltammetry," *Analytica Chimica Acta*, vol. 467, no. 1-2, pp. 179–188, 2002.

[11] R. D. Gerardi, N. W. Barnett, and S. W. Lewis, "Analytical applications of tris(2,2′-bipyridyl)ruthenium(III) as a chemiluminescent reagent," *Analytica Chimica Acta*, vol. 378, no. 1–3, pp. 1–41, 1999.

[12] I. Rubinstein, C. R. Martin, and A. J. Bard, "Electrogenerated chemiluminescent determination of oxalate," *Analytical Chemistry*, vol. 55, no. 9, pp. 1580–1582, 1983.

[13] G. P. Jirka and T. A. Nieman, "Modulated potential electrogenerated chemiluminescence of luminol and $Ru(bpy)_3^{2+}$," *Microchimica Acta*, vol. 113, no. 3–6, pp. 339–347, 1994.

[14] R. J. Forster and C. F. Hogan, "Electrochemiluminescent metallopolymer coatings: combined light and current detection in flow injection analysis," *Analytical Chemistry*, vol. 72, no. 22, pp. 5576–5582, 2000.

[15] R. Wilson, H. Akhavan-Tafti, R. DeSilva, and A. P. Schaap, "Comparison between acridan ester, luminol, and ruthenium chelate electrochemiluminescence," *Electroanalysis*, vol. 13, no. 13, pp. 1083–1092, 2001.

